# Verlorenes Glück — Zufriedenheitsverluste in der Corona-Krise

**DOI:** 10.1007/s10273-020-2715-2

**Published:** 2020-08-21

**Authors:** Michael Ahlheim, Stefan Bruckmeyer, Kai A. Konrad, Lisa Windsteiger

**Affiliations:** 1 Institut für Volkswirtschaftslehre (520F), Universität Hohenheim, Schloss Hohenheim 1, 70599 Stuttgart, Deutschland; 2 und Öffentliche Finanzen, Max-Planck-Institut für Steuerrech, Marstallplatz 1, 80539 München, Deutschland

**Keywords:** D63, I31, J16

## Abstract

Die Corona-Krise betrifft die Bevölkerung vor allem auf der wirtschaftlichen Ebene. Aber auch die psychosozialen und gesellschaftlichen Kosten sind beträchtlich. Der Umfang der Verluste an Lebenszufriedenheit wird auf Basis einer repräsentativen Studie aus dem April 2020 untersucht. Diese Verluste treten vor allem bei Selbständigen auf, insbesondere bei Solo-Selbständigen und Unternehmerinnen, deren Tätigkeit ohnehin mit größeren Unsicherheiten und einer geringeren sozialen Absicherung verbunden ist.

## Abstract (en)

This study illustrates the loss of life satisfaction, and with it the psychological costs of the COVID-19 pandemic and the lockdown measures taken, for self-employed and women. Building on the data collected by Windsteiger et al. (2020) and their internet interviews at the peak of lockdown measures, and looking at specific population subgroups, this essay illustrates that many solo self-employed and women report a significant decline in life satisfaction, and that these effects are strongest where solo self-employment coincides with economic losses and childcare responsibilities for women with children of dependent age.

Wenige Ereignisse haben eine so universelle Betroffenheit ausgelöst wie die COVID-19-Pandemie und die politischen Maßnahmen, die zu ihrer Eindämmung getroffen wurden. Zu den Faktoren gehören neben der gesundheitlichen Gefährdung durch das Virus selbst die Lockdown-Maßnahmen. Bei den damit einhergehenden Kollateralschäden denkt man vielleicht zunächst an den massiven Wirtschaftseinbruch, die gewaltig gestiegenen Staatsschulden und Unsicherheiten darüber, wie Deutschland oder auch ganz Europa aus dieser tiefen Wirtschaftskrise zurückfinden mag.[1]

Psychosoziale und gesellschaftliche Kosten der Pandemie und der ergriffenen Maßnahmen finden geringere Beachtung (unter anderem Hipp und Munnes, 2020; Bertschek und Erdsiek, 2020; Möhring, Naumann, Reifenscheid et al., 2020; Schröder, Entringer, Goebel et al., 2020). Tatsächlich führte die Krise zu erheblichen Beeinträchtigungen des Lebens, etwa im Zusammenhang mit der Schließung von Schulen und Kindertagesstätten, im Zusammenhang mit den Besuchsverboten in Krankenhäusern, Pflege- und Seniorenheimen, in den Beschneidungen vieler Aktivitäten im Alltagsleben, aber auch in Gestalt von Sorgen und Ängsten, von denen nicht alle wirtschaftlicher Natur sind. Als zentrale Messgrößen für diese Lasten bieten sich Maße des subjektiven Wohlbefindens bzw. deren Veränderung an, wie sie in der Glücksforschung seit einigen Jahrzehnten einen erheblichen Aufmerksamkeitszuwachs erfahren haben (Frey und Stutzer, 2002; Helliwell und Putnam, 2004; Clark, Frijters und Shields, 2008; Diener, 2012; Clark, 2018; Diener, Oishi und Tay, 2018).

## Die Daten

Wir nutzen die Antworten von 4.000 Befragten aus einem Internetexperiment für eine Studie von Windsteiger, Ahlheim und Konrad (2020) aus dem April 2020. Diese Studie ist repräsentativ nach Geschlecht, Alter, Haushaltseinkommen und Region und misst die unmittelbaren Folgen für die Lebenszufriedenheit auf dem Höhepunkt der Lockdown-Maßnahmen. Im Zentrum steht die Differenz zwischen der erinnerten Zufriedenheit zu Jahresbeginn und der Zufriedenheit zum Befragungszeitpunkt, gemessen durch die Antworten auf die Frage nach der subjektiven Lebenszufriedenheit auf einer Skala von 1 (überhaupt nicht zufrieden) bis 10 (völlig zufrieden). Die Heterogenität der Befragten in ihren Zufriedenheitsverlusten auf der einen und ihre sozioökonomischen Unterschiedlichkeiten auf der anderen Seite erlauben im Sinne einer Difference-in-Difference-Analyse die Bedeutung der sozioökonomischen Unterschiede für die Zufriedenheitsverluste zu beschreiben.

1 Im Mittelpunkt der Kostendiskussion standen sowohl die durch COVID-19-verursachte Übersterblichkeit und mögliche Überlastungen des Gesundheitssystems als auch die durch den Lockdown verursachte Rezession bzw. Einkommenseinbußen. Über Letztere wurde schon viel geschrieben (z. B. European Commission, 2020; Deutsche Bundesbank, 2020; SVR, 2020).

**Prof. Dr. Michael Ahlheim** ist Fachgebietsleiter am Institut für Volkswirtschaftslehre der Universität Hohenheim.

**Prof. Dr. Kai A. Konrad** ist Direktor am Max-Planck-Institut für Steuerrecht und Öffentliche Finanzen in München. **Dr. Lisa Windsteiger** ist dort wissenschaftliche Referentin. **Stefan Bruckmeyer** ist dort studentischer Mitarbeiter.

Abbildung 1
**Lebenszufriedenheitsverluste von Selbständigen**
Kerndichteschätzung

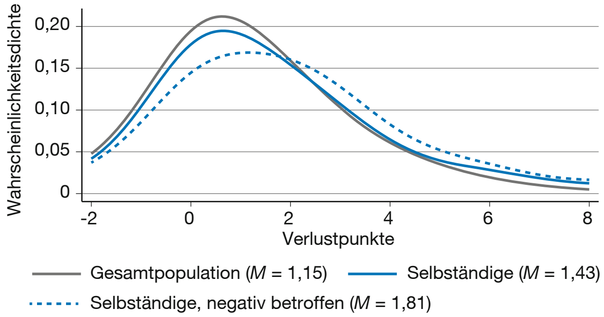

Quelle: eigene Berechnungen.

Abbildung 2
**Lebenszufriedenheitsverluste und Art der Selbständigkeit**
Kerndichteschätzung

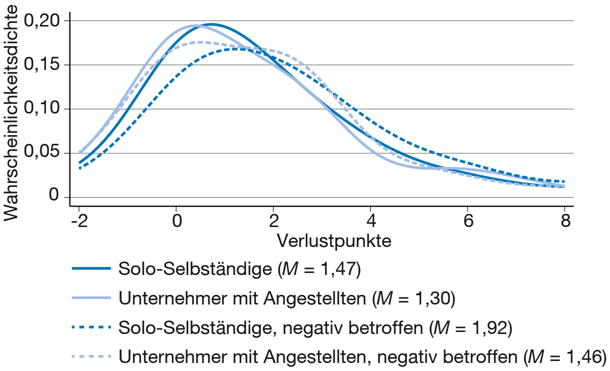

Quelle: eigene Berechnungen.

Windsteiger et al. (2020) hatten das Hauptaugenmerk auf die Frage, wie sich der Zufriedenheitsverlust durch psychologische Charaktereigenschaften wie beispielsweise psychologische Reaktanz[2] erklären lässt, gerichtet. In den multivariaten Schätzungen der Studie, die den Zusammenhang zwischen Reaktanz und Zufriedenheitsverlust untersuchen, wurde eine Reihe von exogenen sozio-ökonomischen Charakteristika als Kontrollvariablen berücksichtigt. Aus diesen Schätzungen ergeben sich bereits erste Indikationen, welche sozioökonomischen Eigenschaften und Lebensumstände den zu erwartenden allgemeinen Einbruch der Zufriedenheitswerte verstärken oder abschwächen. Insbesondere weisen Frauen und Selbständige einen signifikant stärkeren Rückgang der Zufriedenheit auf als der Durchschnitt der Befragten. Ziel des vorliegenden Artikels ist es, diese beiden Betroffenengruppen weiter aufzugliedern.

## Selbständige und Unternehmer insgesamt

Berufliche Selbständigkeit ist dadurch charakterisiert, dass die Betroffenen bei ihrer beruflichen Tätigkeit weniger weisungsgebunden sind. Daraus folgt, dass sie in keine Arbeitshierarchie eingebunden sind und freier über ihre Arbeitszeit verfügen können. Ein Nachteil beruflicher Selbständigkeit ist, dass diese Berufsgruppe im Krisenfall in viel geringerem Umfang Anspruch auf sozialstaatliche Unterstützungsleistungen hat und die finanziellen Risiken ihrer Berufstätigkeit weitgehend selbst tragen muss. Durch die Beschränkungsmaßnahmen aufgrund der COVID-19-Krise wie die Schließung von Geschäften, Freizeiteinrichtungen, Restaurants, kulturellen Einrichtungen etc. wurden Selbständige somit nicht nur wie alle Bürger in ihrem Alltagsleben eingeschränkt, sondern mussten darüber hinaus auch drastische Einkommensverluste bis zum Totalausfall hinnehmen.

Betrachtet man innerhalb der Gruppe der Selbständigen diejenigen, die angaben, dass ihr Unternehmen bzw. Gewerbe infolge der COVID-19-Krise geringere Einnahmen zu verbuchen hatte oder den Betrieb ganz einstellen musste, so ergibt sich im Schnitt eine Einbuße an Lebenszufriedenheit in Höhe von 1,81 Punkten. Diese Zusammenhänge werden auch in Abbildung 1 deutlich, in der die kumulierte Veränderung der subjektiven Lebenszufriedenheit für die gesamte Stichprobe, die Gruppe der Selbständigen und die Teilgruppe der Selbständigen, die COVID-19-bedingt Einkommenseinbußen hinnehmen mussten, dargestellt ist.

Die Unterschiede werden noch dramatischer, wenn man zwischen der Gruppe aller Selbständigen und den Solo-Selbständigen, d. h. Selbständigen ohne weitere Mitarbeiter, unterscheidet. Solo-Selbständige, die infolge der COVID-19-Krise Einkommenseinbußen erlitten, verzeichnen im Durchschnitt einen Rückgang ihrer Lebenszufriedenheit um 1,92 Punkte. Dies wird auch aus Abbildung 2 ersichtlich, wo zwischen Solo-Selbständigen (minus 1,47) insgesamt, Selbständigen mit Mitarbeitern insgesamt (minus 1,30) sowie den infolge der Krise jeweils finanziell negativ betroffenen Solo-Selbständigen (minus 1,92) und Selbständigen mit Mitarbeitern (minus 1,46) unterschieden wird. Man sieht deutlich, dass Selbständige mit Mitarbeitern weitaus weniger unter der Krise leiden als Solo-Selbständige. Dies gilt sowohl für die jeweiligen Gesamtgruppen als auch für den Vergleich der Untergruppen der finanziell negativ betroffenen Selbständigen. Es überrascht nicht, dass die negativ betroffenen Solo-Selbständigen stärkere Zufriedenheits-

2 Psychologische Reaktanz wurde erstmals von Brehm (1966) ausführlich beschrieben. Dabei reagieren Menschen auf Einschränkungen, die ihnen von außen aufgezwungen werden, häufig dadurch, dass sie diese Einschränkungen offen verletzen (z. B. eine Ausgangssperre) oder ihnen zwar gehorchen, sie aber innerlich zutiefst ablehnen und so einen erheblichen Verlust an Lebenszufriedenheit erfahren. In der Studie von Windsteiger et al. (2020) geht es vor allem um die zweitgenannte Form psychologischer Reaktanz, d. h. um den Verlust an Lebenszufriedenheit infolge der COVID-19-bedingten Beschränkungen des Alltagslebens.

verluste berichten als die Gesamtgruppe von Solo-Selbständigen und das entsprechend auch für negativ betroffene Unternehmer mit Angestellten gilt.

Weniger eindeutig ist die Interpretation der höheren Zufriedenheitsverluste von Solounternehmern im Vergleich zu Unternehmern mit Angestellten. Man hätte auch denken können, dass die Krise Unternehmer mit Angestellten stärker trifft, weil das Volumen des finanziellen Verlusts größer ist, weil der Niedergang eines traditionsreichen Familienunternehmens für den Unternehmer mit einem großen Gesichtsverlust verbunden ist oder er es als Versagen seinen Arbeitnehmern gegenüber empfindet. Anscheinend wiegen indes Betroffenheitsaspekte bei den Solo-Unternehmern schwerer. Vielleicht hilft in Betrieben mit mehreren Mitarbeitern das Gemeinsamkeitsgefühl in der Krise, das Solo-Unternehmer so nicht erleben. Vielleicht unterscheiden sich die Gruppen auch in ihren Erfahrungen, was vorherige Krisen angeht.

Die finanziellen Auswirkungen der Corona-bedingten Beschränkungsmaßnahmen unterscheiden sich ebenfalls, sodass auch handfeste finanzielle Gründe zu einer größeren Betroffenheit von Solo-Selbständigen führen mögen. Sie können zwar, wie alle Selbständigen mit bis zu fünf Mitarbeitern, im Rahmen der Corona-Soforthilfen der Bundesregierung und der Länder einmalige nichtrückzahlbare Zuschüsse in Höhe von maximal 9.000 Euro beantragen (bei Selbständigen mit sechs bis zehn Mitarbeitern sind es maximal 15.000 Euro), aber diese Zuschüsse dürfen nur zur „Sicherung ihrer wirtschaftlichen Existenz und der Überbrückung von akuten Liquiditätsengpässen“ verwendet werden. „Dagegen können Kosten des privaten Lebensunterhalts wie die Miete der Privatwohnung oder Krankenversicherungsbeiträge nicht durch die Soforthilfe abgedeckt werden.“ (BMWi, 2020). Für derartige Aufwendungen werden in finanzielle Not geratene Solo-Selbständige auf Leistungen nach dem Sozialgesetzbuch II, insbesondere dem Arbeitslosengeld II, verwiesen. Ein Problem vieler Solo-Selbständiger kann darin bestehen, dass sie in Zeiten, in denen sie nicht arbeiten können, nur sehr geringe Betriebskosten haben, weil sie ihren Beruf beispielsweise von ihrer Privatwohnung aus oder mobil von verschiedenen Standorten aus ausüben (Briegleb, 2020).

In einer kürzlich erschienenen Studie zur Reaktion von Solo-Selbständigen auf die COVID-19-Krise fanden Block et al. (2020), dass ein Großteil der Solo-Selbständigen mit einem Alter von über 60 Jahren inzwischen ans Aufgeben denken. Auch Bertschek und Erdsiek (2020) konstatieren auf Basis einer umfragegestützten Studie des ZEW in Mannheim eine desolate Situation der Solo-Selbständigen: „Jeder Vierte der befragten Soloselbstständigen sieht eine hohe Wahrscheinlichkeit dafür, die eigene Selbstständigkeit in den nächsten zwölf Monaten aufgeben zu müssen. … Bei knapp 60 % der Befragten ist zum Zeitpunkt der Umfrage im April 2020 der monatliche Umsatz um mehr als 75 % eingebrochen. Aufgrund der notwendigen Kontaktbeschränkungen und Shutdown-Maßnahmen konnte jeder zweite befragte Soloselbstständige seine Tätigkeit zum Zeitpunkt der Umfrage überhaupt nicht mehr ausüben.“ Dass eine solche Situation auf die Lebensfreude drückt, ist leicht nachzuvollziehen.

Abbildung 3
**Lebenszufriedenheitsverluste von Frauen**
Kerndichteschätzung

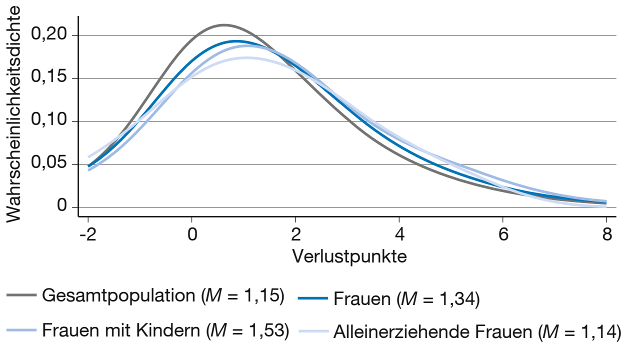

Quelle: eigene Berechnungen.

## Geschlechterunterschiede, Kinderbetreuung und traditionell geprägte Ehemuster

Vor einem Blick auf die Betroffenheitsunterschiede zwischen Unternehmerinnen und Unternehmern blicken wir auf die unterschiedliche Betroffenheit von Frauen über alle Beschäftigungskategorien hinweg. Eine Rolle könnte spielen, ob eine Frau allein oder mit Partner/Partnerin in einem Haushalt wohnt, ob in dem Haushalt Kinder unter 14 Jahren leben und ob die Frau erwerbstätig ist oder nicht.

Abbildung 3 zeigt die Verteilung der Lebenszufriedenheitsverluste für die Gesamtbevölkerung, für die Gruppe der Frauen, für die Gruppe der Frauen mit einem oder mehreren Kindern unter 14 Jahren und für die Teilgruppe unter diesen, die nicht in einem Haushalt mit mehreren Erwachsenen leben. Bei Berücksichtigung der gesamten Stichprobe ergibt sich ein Mittelwert von 1,15. Frauen erfahren hierbei durchschnittlich einen Verlust an Lebenszufriedenheit von 1,34. Für die Teilpopulation der Frauen mit Kindern stellt sich ein Mittelwert von 1,53 ein. Alleinerziehende Frauen weisen einen durchschnittlichen Rückgang der Lebenszufriedenheit von 1,14 Punkten auf.

Zu vermuten ist, dass diese Effekte mit der De-facto-Arbeitsteilung innerhalb vieler Ehen und Familien zusammenhängen. Empirische Befunde zur Arbeitsteilung weisen darauf hin, dass Mütter einen deutlich größeren Anteil an der Kindesbetreuung haben (Bianchi, 2000; Tamm, 2019; Sofer und Thibout, 2019). Diese innereheliche Arbeitsteilung ist wohl nicht nur durch soziale Normen und Pfadabhängigkeiten geprägt. Die Familienökonomie beschreibt sie vielmehr als Ergebnis von kooperativen Verhandlungen innerhalb der Familie, in denen Unterschiede und komparative Vorteile von Mann und Frau auf dem Arbeitsmarkt eine Rolle spielen.[3] Die innereheliche Zuordnung von Betreuungsleistungen, die Erwerbstätigkeit der Eheleute und der Umfang der Nutzung externer Kinderbetreuungseinrichtungen war insofern im Vorkrisen-Normalzustand das Gleichgewichtsergebnis einer Verhandlung unter den bestehenden Rahmenbedingungen und die subjektiven Zufriedenheitswerte vor der Krise reflektierten dieses Gleichgewicht. Durch die Schließung von Kindertagesstätten und Schulen verändern sich die Rahmenbedingungen. Staatliche Betreuungsleistungen entfallen. Die ökonomische Logik einer Familie mit Arbeitsteilung legt nahe, dass diese Veränderungen zunächst zulasten der Person gehen, die sich auch vor diesen Schließungen stärker auf Kinderbetreuungs- und Erziehungsleistungen konzentriert hat und bei diesen Personen zu stärkeren Belastungen führen. Diese Personen sind häufiger die Mütter als die Väter. Diese Kriseneffekte sind quantitativ bedeutend und mögen erschrecken. Verfrüht, und vermutlich auch unbegründet wäre es indes, aus dem Krisenbefund zu schließen, dass sich diese Verteilungswirkungen und Kriseneffekte für die zukünftige Arbeitsteilung in der Ehe verfestigen und die Corona-Krise überdauern.

## Selbständige Frauen und Unternehmerinnen

Unterscheidet man innerhalb der Gruppe der Selbständigen zwischen den Geschlechtern, so zeigt sich, dass Frauen mit einem durchschnittlichen Verlust an Lebenszufriedenheit in Höhe von 1,85 Punkten härter von der Krise betroffen sind als der Durchschnitt aller Selbständigen (minus 1,43). Innerhalb dieser Gruppe verzeichnen vor allem Frauen, die angeben, negativ durch die Corona-Maßnahmen betroffen zu sein, einen dramatischen Einbruch ihrer Lebenszufriedenheit mit minus 2,29 Punkten. Letztlich addieren sich für diesen Personenkreis drei negative Kriseneffekte, die für sich und einzeln betrachtet bereits in den multivariaten Analysen von Windsteiger et al. (2020) als signifikante Einflussfaktoren identifiziert werden konnten (vgl. Abbildung 4).

Mögliche Ursachen lassen sich mehrere erahnen. Zunächst könnten sich die Nachteile des Selbständigseins mit den Nachteilen aus der familiären Rolle bei der Kindererziehung addieren. Ferner könnten auch andere Faktoren eine Rolle spielen. Die Studie von Block et al. (2020) zeigt z. B., dass

3 Bei diesen Verhandlungen spielen Drohpunkte (z. B. nicht-kooperative Gleichgewichte der Aufgaben-, Einkommens- und Arbeitsteilung und die Ausbildungsniveaus der Eltern) eine entscheidende Rolle (Theoretische Arbeiten z. B. Lundberg und Pollak, 1993; Konrad und Lommerud, 2000; Lommerud, Straume und Vagstad, 2015; Meier und Rainer, 2017).

Abbildung 4
**Lebenszufriedenheitsverluste von selbständigen Frauen**
Kerndichteschätzung

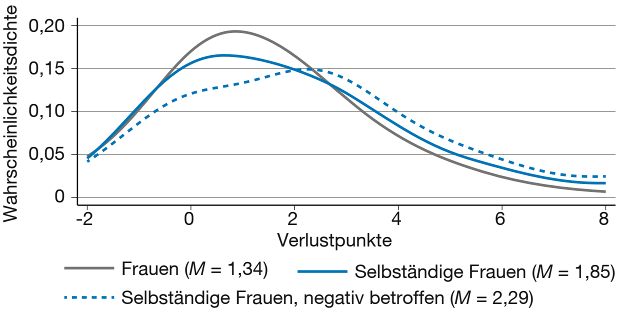

Quelle: eigene Berechnungen.

solo-selbständige Frauen signifikant weniger häufig zu Gegenmaßnahmen wie finanziellem Bootstrapping (Gründung ohne externe finanzielle Unterstützung) greifen, um die Liquidität ihres Unternehmens zu sichern, und damit eher von Insolvenz bedroht sind als ihre männlichen Kollegen. Dies wiederum könnte mit den familiären Zwängen in der Kinderbetreuung, Home Schooling etc. verbunden sein. So hätten sie weniger Zeit, um sich um Möglichkeiten für zusätzliche staatliche Hilfen zu kümmern oder sich über Strategien zur Sicherung ihrer Liquidität zu informieren und die dann unter Umständen auch anstehenden Auseinandersetzungen mit Lieferanden, Kunden, Vermietern etc. zu führen.

## Schlussfolgerungen

Insgesamt offenbart die Studie, dass die psychologischen Kosten der COVID-19-Krise bei Selbständigen deutlich stärker ausgeprägt sind als im Durchschnitt der Bevölkerung und dass innerhalb dieser Gruppe Solo-Selbständige und selbständig tätige Frauen besonders hart von Verlusten an Lebenszufriedenheit getroffen sind. Diese psychologischen Kosten der COVID-19-Krise berücksichtigen zwar die finanziellen Einbußen der verschiedenen Bevölkerungsgruppen, aber sie gehen darüber hinaus, weil sie auch die in die Zukunft gerichteten Ängste der Betroffenen und ihre tiefe Verunsicherung und die Veränderungen der sozialen und familiären Lebensumstände berücksichtigen. Daher ergänzt die Messung dieser Kosten der Krise mit Hilfe einer Lebenszufriedenheitsanalyse die Studien zur Erfassung der rein finanziellen Folgen in zweierlei Hinsicht. Zum einen werden auch nicht-finanzielle Effekte der Krise erfasst, die durchaus erheblich sind, wie unter anderem die Demonstrationen der letzten Zeit gezeigt haben, und zum andern wird die objektiv messbare Maßeinheit Geld durch das subjektive Maß der selbst-empfundenen Lebenszufriedenheit ergänzt. Dieser sollte sowohl aus wohlfahrtsökonomischer als auch aus politischer Sicht das Hauptaugenmerk einer Regierung gelten, wobei finanzielle Aspekte nur einen Teilaspekt darstellen.

Natürlich sind die finanziellen Folgen der Krise von erheblicher Bedeutung für die Gesamtbevölkerung, aber insbesondere auch für Selbständige und Frauen. So stimmen in der Studie von Windsteiger et al. (2020) rund 55 % aller Selbständigen der Aussage „Ich fürchte große negative finanzielle Konsequenzen in der Zukunft“ entweder „voll zu“ oder „eher zu“, während dies im Durchschnitt aller Befragten nur für 42 % der Probanden gilt. Hier zeigt sich, dass die von der Regierung bereitgestellten Corona-Hilfen die finanziellen Kosten bei den Selbständigen nur teilweise kompensieren und dass die psychologischen Kosten vor allem auch mit der individuell empfundenen Vulnerabilität der Betroffenen korreliert sind. So sind Selbständige schlechter für die Krise gewappnet als der Durchschnitt der Bevölkerung und sie sind sich dessen offensichtlich auch bewusst. Die Solo-Selbständigen sind noch verwundbarer als der Durchschnitt aller Selbständigen und auch dies spiegelt sich in ihrem entsprechend größeren Verlust an Lebenszufriedenheit wider. Ferner ergeben sich bedeutende Geschlechterunterschiede. Frauen sind stärker betroffen, besonders solche mit Kindern im betreuungsintensiven Alter. Diese Unterschiede gelten insbesondere auch für selbständige Frauen und Unternehmerinnen.

Für die Bewertung der Größenordnung dieser Einbußen kann man Vergleiche mit dem Durchschnitt des Rückgangs an Lebenszufriedenheit in der Folge von Schicksalsschlägen heranziehen. Zu Vergleichswerten sei auf Felbermayr et al. (2017, 22) für Daten aus dem GSÖP verwiesen. Diese berichten Rückgänge der Lebenszufriedenheit durch Arbeitslosigkeit von 0,49 Punkten, für Alleinlebende von 0,20, Verwitwete von 0,31, Geschiedene von 0,08. Dies illustriert die erhebliche Größenordnung der in Windsteiger et al. (2020) ermittelten Verluste an Lebenszufriedenheit infolge der Corona-Krise.

**Title:** ***Lost Happiness – Loss of Life Satisfaction in the Corona Crisis***

**JEL Classification:** *D63, I31, J16*
